# Nearly a century of discoveries in bacterial genetics and their continuing impact on the field

**DOI:** 10.1093/g3journal/jkaf225

**Published:** 2025-11-12

**Authors:** David A Baltrus, Houra Merrikh, Hildegard Uecker, Alex Wong

**Affiliations:** School of Plant Sciences, University of Arizona, Tucson, AZ, 85721, United States; Department of Biochemistry, Vanderbilt University School of Medicine, Nashville, TN, 37235, United States; Department of Evolutionary Theory, MPI for Evolutionary Biology, Plön 24306, Germany; Department of Biology, Carleton University, Ottawa, ON K1S 5B6, Canada

## Abstract

With this special issue on the Genetics of Bacteria, the authors shine new light on selections and screens from the past (and on the people that carried these out) while also pointing towards many future directions for bacterial genetics. Their goal is to highlight the GSA journals as a welcome home for reporting discoveries across bacterial systems building on their rich history.

The Genetics Society of America has a rich tradition of supporting important bacterial genetics works, with numerous foundational papers published in the journal Genetics. These include elegant and groundbreaking experiments, such as publication of the original fluctuation tests by Luria and Delbrück and characterization of lysogens by the Lederbergs (Luria and Delbrück 1943; Lederberg and Lederberg 1953), which informed our understanding of genetics across all of biology. In addition, there were many others which not only demonstrated that bacteria were more complex than “bags of enzymes” but could themselves be powerful models to tease apart how life works (Demerec and Fano 1945; Lederberg 1947; Witkin 1947; Luria and Dulbecco 1949; Lieb 1951; Falkow et al. 1961). There have been incredible technological advances since the early days of *E. coli* and lambda phage, developments which have revolutionized research across all fields. Such advances become powerful accelerators of genetic discovery when coupled with useful characteristics of microbial systems like large population sizes, rapid growth, and ease of genetic manipulation. Today, automation of laboratory passage experiments combined with cost efficient whole genome sequencing methods is enabling us to precisely identify single nucleotide changes underlying phenotypes of interest within populations and across communities (Ascensao and Desai 2025). Moreover, combining elegant design screens and selections with development of tools like fluorescent markers and cryo-EM can provide transformative insights into microbial physiology and structure (Frei et al. 2024; Nogales and Mahamid 2024). With this special issue on the Genetics of Bacteria, we shine new light on selections and screens from the past (and on the people that carried these out) while also pointing toward many future directions for bacterial genetics. Our goal is to highlight the GSA journals as a welcome home for reporting discoveries across bacterial systems building on this rich history.

## Mutation as the driver of bacterial genetics discoveries

Experiments from Luria and Delbrück published in Genetics were critical for establishing that mutation could be studied in both quantitative and qualitative terms, for showing how researchers from across disciplines could collaborate and inform each others' viewpoints in new and exciting ways, and for demonstrating how large population sizes of *Escherichia coli* facilitated new studies into the quantification of mutation rates (Luria and Delbrück 1943). A review from Lovett and Sass (2025) in this issue describes the elegance and importance of the Luria and Delbrück experiment in the context of contemporaneous studies while highlighting how our understanding of mutation in bacterial systems has grown and matured over the last 8 decades.

Although studies of mutation rates ushered in the modern era of bacterial genetics, the ease with which large bacterial populations acquire new diversity through mutations have enabled countless discoveries throughout microbiology, genetics, and evolutionary biology since the time of Luria Delbrück. Following this tradition, an article from Jena et al. (2025) in this issue highlights how mutations arising during laboratory evolution studies point toward different and unique paths of adaptation to sublethal levels of antibiotics.

## The power of recombination to shape microbial populations

While mutation introduces genetic variation into microbial populations, recombination and horizontal gene transfer can move this variation across genetic backgrounds and species. Just as Luria and Delbrück introduced the power of selection and mutagenesis for bacterial genetics, the pioneering experiments of the Lederbergs gave the world its first glimpse that this variation could move throughout bacteria and documented the mechanisms of such transfers (Lederberg 1947; Lederberg and Lederberg 1953). Although Joshua receives extensive credit for this work, including a Nobel Prize, the accomplishments by his wife (at the time) Esther have not had as much of a spotlight shined on them. Here, Wendling and Bailey (2025) provide a look into critical roles that Esther played for many incredible discoveries in the early days of bacterial genetics from conjugation of *E. coli* to Lambda phage to the innovation of replica plating.

The realization that bacteria could exchange genetic information with each other spawned numerous investigations to take account of the evolutionary importance of such transfers across populations. However, given difficulties in predicting recombination and horizontal gene transfer throughout bacterial taxa prior to the era of genomics, many questions remain unanswered about the frequency of transfers within and between populations and communities under natural conditions as well as how this process could shape bacterial speciation events. Enabled by the recent accumulation of thousands of bacterial genomes, in this issue, Preska Steinberg and Kussell (2025) analyze recombination of single nucleotide polymorphisms across multiple bacterial species to more clearly demonstrate how widespread recombination shapes allelic diversity of bacteria, providing data facilitating predictions for the power of such events to shape future bacterial evolution.

One can also leverage recombination events across strains to identify the genetic basis of phenotypes of interest through genome-wide association studies (GWAS). Since qualitative phenotypic changes in bacteria are often driven by presence-absence polymorphisms, GWAS studies can be particularly powerful tools to pinpoint causative genetic variants if whole genomes are available. This is especially so if phenotypic changes occur frequently and independently throughout branches of a phylogeny. In this special issue, Baltrus et al. (Baltrus et al. 2025 ) use GWAS pipelines to uncover recombination-driven evolutionary changes in lipopolysaccharide biosynthesis pathways which drive differential sensitivity to strain specific antimicrobials like tailocins.

Lastly, enzyme complexes like RecBCD are essential for facilitating recombination events within populations and across strains and species. However, given that RecBCD has nuclease functions which in turn can facilitate RecA loading, lore exists throughout past literature that RecBCD could implicitly function in bacterial defense against lytic phage by destroying phage genomes prior to their replication. Zheng et al. (Zheng et al. 2025) provide direct data to refute this claim by demonstrating that there are no differences between wild-type and *recBCD-* strains in their ability to host phage from a variety of environmental sources.

## Leveraging genetics and theory to enable future technologies

Bacterial genetics is at the origin of the revolutionary CRISPR-Cas technology, which has numerous important applications in medicine and agriculture (Jinek et al. 2012; Wang and Doudna 2023). Although still early days for the technology, it is already successfully applied to treat genetic diseases (Parums 2024). One potential further application is the use of CRISPR-Cas–derived tools to sensitize antibiotic-resistant bacteria (Benz et al. 2025). However, besides technical obstacles, 1 limitation of this approach is that mutation of CRISPR target sequences could enable bacterial escape from the CRISPR treatment. Theoretical modeling can help to obtain a clear picture of this risk even before the technology is ready. Taking potential CRISPR-Cas evading mutations into account, Kippnich et al. (2025) develop a proof-of-principle model to compare the potential of various CRISPR treatment strategies in sensitizing a bacterial population with plasmid-borne resistance for subsequent antibiotic treatment.

The articles in this special issue highlight both the significant historical accomplishments of bacterial genetics, as well as recent developments and technologies. Themes struck by Luria, Delbrück, Esther, and Joshua Lederberg and other pioneers still motivate the field today, but with variations unforeseeable in the 1940s and 1950s. We look forward to the achievements of the next 80 years.
